# Peritoneal dialysis for COVID-19-associated acute kidney injury

**DOI:** 10.1186/s13054-020-03024-z

**Published:** 2020-06-08

**Authors:** Mika Nagatomo, Hiroyuki Yamada, Ken Shinozuka, Manabu Shimoto, Tomoyuki Yunoki, Shigeru Ohtsuru

**Affiliations:** 1 Department of Primary Care and Emergency Medicine, Graduate School of Medicine, Kyoto University, 54 Shogoin-kawaharacho, Sakyo-ku, Kyoto, 6068507 Japan; 2 Department of Nephrology, Graduate School of Medicine, Kyoto University, Kyoto, Japan

Dear Editor,

Coronavirus disease 2019 (COVID-19) is rapidly spreading all over the world. One of the reasons for this quick spread is that the virus may be transmitted via aerosolize particles from asymptomatic individuals. To avoid further transmission, a huge amount of personal protective equipment (PPE) and specialized medical equipment is necessary. As the pandemic evolves, the shortage of these resources has become one of the biggest social problems worldwide. Thus, we are forced to provide appropriate treatments with limited available resources.

Acute kidney injury (AKI) is also one of the significant complications of COVID-19, along with respiratory failure [1]. Recent retrospective studies have shown that COVID-19 patients who develop acute kidney injury (AKI) have an extremely poor prognosis [2–4]. To rescue these severe patients, renal replacement therapy (RRT) should definitely be considered. However, in terms of infection control and medical resources, it is much more challenging to perform hemodialysis than before the pandemic happened. Herein, using an actual case, we argue that peritoneal dialysis (PD) could become a more practical and safer RRT, particularly during this pandemic crisis.

A 62-year-old male with a PCR test positive for severe acute respiratory syndrome coronavirus 2 (SARS-CoV-2) developed dyspnea. After admission to the depressurized room in our intensive care unit (ICU), his oxygenation and hemodynamics rapidly deteriorated. We started mechanical ventilation and administered vasopressors favipiravir and ciclesonide. Additionally, serum creatinine became elevated (day 1 0.77 mg/dL→day5 5.19 mg/dL), and his urine volume also dropped to 0.1 mL/kg/day; we diagnosed him with COVID-19-associated AKI. As he progressed to anuria, it became difficult to control the serum potassium and the hemodynamics due to acidemia. We inserted the PD catheter to the recto-vesical pouch using a portable X-ray machine at the bedside and infused the peritoneal dialysate (Fig. 1a–c). Although the anuria persisted for a while, the increased amount of peritoneal dialysate easily normalized the acidemia and serum potassium level. Also, the PD procedure did not influence the patient’s hemodynamics or respiratory status. After the normalization of acidemia and electrolytes, the vasopressors could be tapered off, and the inflammation status was also improved. On day 14, the patient was discharged from our ICU with the PD catheter. SARS-CoV-2 was detectable in the sputum, but not in the peritoneum and PD waste.

**Fig. 1:**
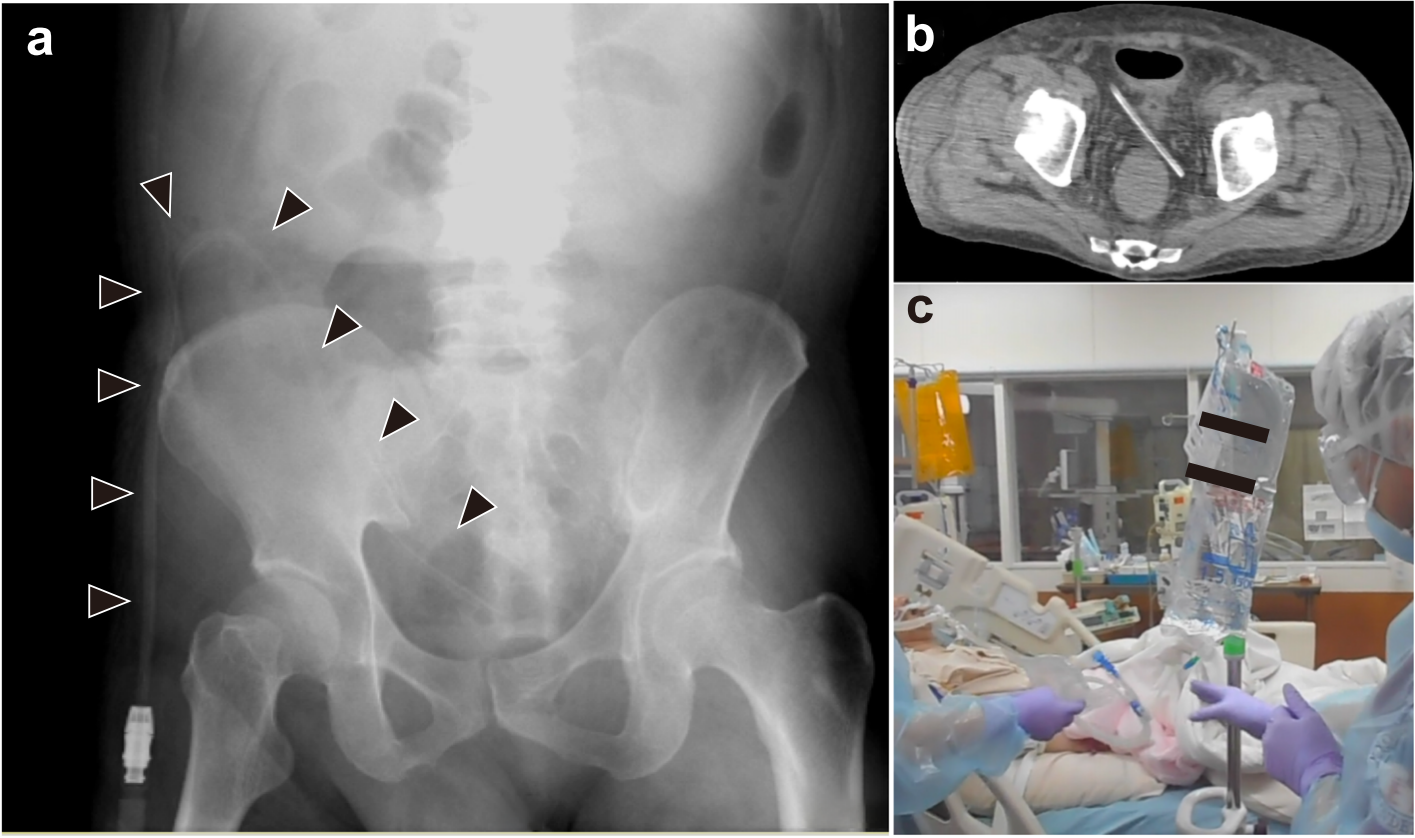
**a** Abdominal X-ray of peritoneal dialysis (PD) catheter. Arrowhead indicates the PD catheter. **b** The PD catheter tip confirmed in the recto-vesical pouch by CT scan image taken after ICU discharge. **c** Actual condition of peritoneal dialysis for COVID-19 intubated patients

Generally, before the COVID-19 pandemic, continuous renal replacement therapy (CRRT) was the first choice for RRT in hemodynamically unstable patients [5]. However, after the outbreak, it has become difficult to allocate hemodialysis machines to COVID-19 patients with AKI due to infection control [6]. Additionally, CRRT requires continuous health provider’s care and troubleshooting such as changing anticoagulants, correcting poor blood drainage, and responding to various sudden alarms. It tragically wastes PPE and further exposes medical staff to SARS-CoV-2 infection risk.

Meanwhile, PD does not require specialized instruments, and the procedure of peritoneal dialysate exchange is quite simple. It could also improve the electrolyte and acid/base balance similarly as hemodialysis, except for water removal. Furthermore, as this case has shown, the insertion of the PD catheter is possible even at the bedside. Therefore, PD could be applicable to various treatment situations for severe COVID-19 patients, including those in developing countries. Surely, periodic dialysate exchange might become indispensable at any treatment phase. However, since the timing of exchange could be controllable, we could exchange it at the same time as other procedures, such as infusion bottle exchange and postural changes. This does not lead to more waste of PPE or more explosion of medical staff to COVID-19 infection. Thus, as PD relatively matches with social and technical needs more than before the pandemic, it should be positively taken into consideration in this global crisis.

## Data Availability

The datasets from this study are available from the corresponding author on request.

## References

[1] Fanelli V, Fiorentino M, Cantaluppi V, Gesualdo L, Stallone G, Ronco C, Castellano G (2020). Acute kidney injury in SARS-CoV-2 infected patients. Crit Care (London).

[2] Chen T, Wu D, Chen H, Yan W, Yang D, Chen G, Ma K, Xu D, Yu H, Wang H (2020). Clinical characteristics of 113 deceased patients with coronavirus disease 2019: retrospective study. BMJ.

[3] Cheng Y, Luo R, Wang K, Zhang M, Wang Z, Dong L, Li J, Yao Y, Ge S, Xu G (2020). Kidney disease is associated with in-hospital death of patients with COVID-19. Kidney Int.

[4] Deng Y, Liu W, Liu K, Fang YY, Shang J, Zhou L, Wang K, Leng F, Wei S, Chen L, et al. Clinical characteristics of fatal and recovered cases of coronavirus disease 2019 (COVID-19) in Wuhan, China: a retrospective study. Chin Med J. 2020. 10.1097/CM9.0000000000000824. 10.1097/CM9.0000000000000824 PMC7289311 32209890

[5] KDIGO AKI Working Group (2012). KDIGO clinical practice guideline for acute kidney injury. Kidney Int Suppl.

[6] Goldfarb DS, Benstein JA, Zhdanova O, Hammer E, Block CA, Caplin NJ, Thompson N, Charytan DM. Impending Shortages of Kidney Replacement Therapy for COVID-19 Patients. Clin J Am Soc Nephrol. 2020. 10.2215/CJN.05180420. 10.2215/CJN.05180420 PMC7274293 32345750

